# Microstructure-Based Fiber-To-Chip Coupling of Polymer Planar Bragg Gratings for Harsh Environment Applications

**DOI:** 10.3390/s20195452

**Published:** 2020-09-23

**Authors:** Stefan Kefer, Theresia Sauer, Steffen Hessler, Michael Kaloudis, Ralf Hellmann

**Affiliations:** 1 Applied Laser and Photonics Group, Aschaffenburg University of Applied Sciences, Wuerzburger Strasse 45, 63743 Aschaffenburg, Germany; steffen.hessler@th-ab.de (S.H.); ralf.hellmann@th-ab.de (R.H.); 2 Laboratory for Packaging and Interconnection Technology, Aschaffenburg University of Applied Sciences, Wuerzburger Strasse 45, 63743 Aschaffenburg, Germany; theresiasauer@gmx.de (T.S.); michael.kaloudis@th-ab.de (M.K.)

**Keywords:** Bragg grating sensors, cyclic olefin copolymers, fiber-to-chip coupling, microstructures, micromilling

## Abstract

This article proposes and demonstrates a robust microstructure-based fiber-to-chip coupling scheme for planar Bragg grating devices. A polymer planar Bragg grating substrate is manufactured and microstructured by means of a micromilling process, while the respective photonic structures are generated by employing a sophisticated single-writing UV-exposure method. A stripped standard single-mode fiber is inserted into the microstructure, which is filled with a UV-curable adhesive, and aligned with the integrated waveguide. After curing, final sensor assembly and thermal treatment, the proposed coupling scheme is capable of withstanding pressures up to 10 bar, at room temperature, and pressures up to 7.5 bar at an elevated temperature of 120 °C. Additionally, the coupling scheme is exceedingly robust towards tensile forces, limited only by the tensile strength of the employed single-mode fiber. Due to its outstanding robustness, the coupling scheme enables the application of planar Bragg grating devices in harsh environments. This fact is underlined by integrating a microstructure-coupled photonic device into the center of a commercial-grade carbon fiber reinforced polymer specimen. After its integration, the polymer-based Bragg grating sensor still exhibits a reflection peak with a dynamic range of 24 dB, and can thus be employed for sensing purposes.

## 1. Introduction

The impact of Bragg grating-based optical sensors and sensor systems on current scientific research, as well as in the outline of industrial applications, has grown tremendously within the last decades. In particular, fiber Bragg gratings (FBG) have found their way into numerous commercial fields, such as structural monitoring, oil or gas processing and medical industry branches [1,2,3,4,5]. While most FBG devices are based on silica, current research is heavily involved in the exploration and utilization of polymer-based materials. Besides omitting the necessity of additional doping or hydrogen-loading processes, due to their inherent photosensitivity, polymer fiber Bragg gratings (POF-BGs) offer increased temperature sensitivity as well as a superior strain sensing performance and range, due to their outstanding thermo-optic coefficient and reduced Young’s modulus [6,7].

Another promising approach to polymer-based Bragg grating sensors is found in employing planar substrates. In general, polymer planar Bragg gratings (PPBG) provide the same advantages as POF-BGs, while they are additionally easy to handle and enable the utilization of mass-produced injection-molded substrates. Moreover, they offer straightforward multidimensional strain sensing by monitoring multiple photonic structures on one single substrate [8,9]. To date, a variety of polymer-optic materials, such as poly(methyl methacrylate) (PMMA) [10,11,12], hybrid organic-inorganic polymers [13,14,15], epoxy-based photoresists [16,17,18,19] and cyclic olefin copolymers (COC), Reference [20] can be employed to fabricate PPBGs. The latter in particular has shown unambiguous potential in a multitude of sensing applications, since this high-grade optical polymer exhibits a unique combination of an outstanding glass transition temperature, up to 250 °C, and negligible water absorption [21,22]. Due to their extraordinary material properties, COC-based PPBGs (COC-PPBGs) are capable of performing temperature measurements up to 160 °C, with a sensitivity of −7.3 pm K^−1^ [23], and strain sensing with a sensitivity of 1.2 pm µε^−1^ [24]. Furthermore, as of now, they are the only polymer-based Bragg grating sensor able to withstand the integration process into a commercial-grade carbon fiber reinforced polymer, where the PPBG is exposed to a pressure of 7 bar and a temperature of 120 °C, for a duration of two hours [25]. Moreover, in combination with functional coatings, COC-PPBGs have shown distinct potential in refractive index and hydrogen sensing applications [26,27].

**Figure 1:**
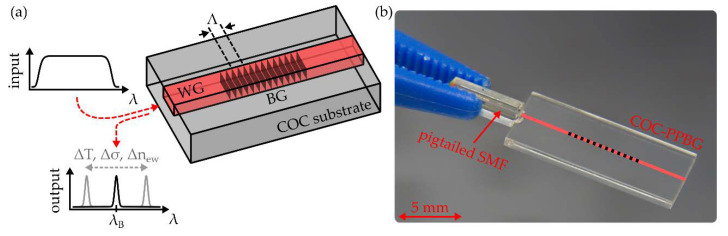
(**a**) Working principle of a polymer planar Bragg grating (PPBG) comprising a buried waveguide (WG) as well as a Bragg grating (BG); (**b**) PPBG based on a cyclic olefin copolymer (COC) substrate. For evaluation purposes, the PPBG needs to be butt coupled to a pigtailed single-mode fiber (SMF).

In principal, COC-PPBGs comprise a buried waveguide, featuring a periodic refractive index perturbation denoted as Bragg grating, as shown in Figure 1a.

The integrated Bragg grating acts as a wavelength selective reflector, whereas its main wavelength of reflection, or Bragg wavelength λ_B_, is defined by the grating’s effective refractive index n_eff_ and its spatial periodicity Λ, according to

λ_B_ = 2n_eff_(ΔT,Δσ,Δn_ew_)Λ(ΔT,Δσ). (1)

Both parameters, and therefore the resulting Bragg wavelength, are eminently susceptible to environmental influences, such as changes in temperature, ΔT, and/or strain, Δσ, whereas the grating’s overall refractive index also includes refractive index changes of the evanescent field, Δn_ew_. Thus, as in the case of FBGs, evaluation of the Bragg wavelength provides accurate information about the intended physical quantity [28].

However, for the monitoring and evaluation of any PPBG’s Bragg wavelength, a physical connection to an interrogation unit needs to be established. This is commonly achieved by butt coupling a pigtailed single-mode fiber (SMF) to the integrated waveguide by means of a UV-curable adhesive, as depicted in Figure 1b. Though sufficiently stable fiber-to-chip connections have been realized for several undemanding applications, this junction is considerably sensitive towards external forces, and thus limits or even prevents the practical harsh environment applicability of planar waveguide sensors in general [29]. Against this background, this study features an experimental examination of the lateral alignment requirements for butt coupled COC-PPBGs. Furthermore, a novel microstructure-based fiber-to-chip coupling scheme for PPBGs is proposed and demonstrated. Due to its outstanding robustness, the optical interconnection is able to withstand temperatures up to 120 °C, pressures up to 10 bar and tensile forces up to the breaking point of the employed silica fiber. Additionally, to highlight and exemplify the rigidity of the established junction, a harsh environment application is presented by fully integrating a PPBG into the center of a commercial-grade carbon fiber reinforced polymer specimen.

## 2. Device Fabrication

Injection-molded COC (TOPAS 6017S-04, TOPAS Advanced Polymers, Raunheim, Germany) plates are microstructured and cut to a rectangular shape by means of a desktop mill (CNC Mini-Mill/4, Minitech Machinery, Norcross, GA, USA). Every sensor consists of two single platelets, where one of them is the designated sensor substrate and the other is used as a lid to protect the fiber-to-chip interface from environmental influences. Besides a T-shaped microstructure, exhibiting a width of 300 μm and a depth of 200 μm, the sensor substrate and lid are both equipped with uniformly distributed indentations or knobs, respectively. An overview of the employed tools as well as the milling parameters used during macro- and microstructuring is given in Table 1.

Subsequently, a waveguide as well as an integrated Bragg grating are simultaneously generated within the sensor substrate by means of the single-writing-step (SWS) procedure [30]. During the process, the sensor substrate is exposed to pulsed UV radiation generated by a krypton fluoride laser (BraggStar Industrial, Coherent Europe B.V., Utrecht, Netherlands), while covered with a phase mask and an amplitude mask at the same time. The former exhibits a grating period of 1020 nm over a length of 10 mm, while the latter has a slit width of 27 μm. The polymer is exposed to multiple pulses with a single pulse energy of 68 μJ and a pulse duration of 15 ns, at a repetition rate of 200 Hz, which results in an overall fluence of 100 J cm^−2^. Afterwards, the sensor substrate is thermally treated for 2 h at a temperature of 130 °C. Photonic structures generated this way generally exhibit a graded-index depth profile and indistinct boundaries. Therefore, they are commonly referred to as diffused waveguides [31]. Please note that an extended study of the SWS procedure, its parameters and their influence on the resulting PPBG’s properties is given by the authors elsewhere [32].

**Figure 2:**
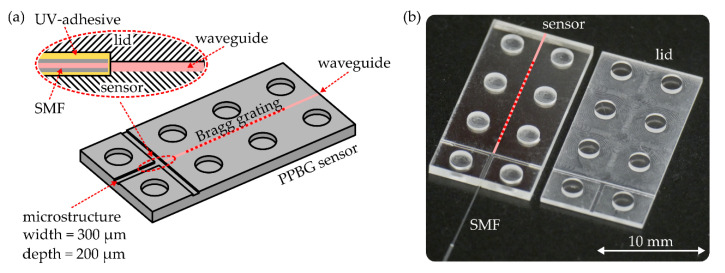
(**a**) Schematic of the microstructured sensor substrate. The zoom in (upper left) depicts a cross section of the final fiber-to-chip coupling configuration. (**b**) Photograph of the microstructured polymer planar Bragg grating (PPBG) sensor butt coupled to a single-mode fiber. The associated lid is subsequently mounted on top of the sensor substrate.

The vertically cleaved end of a standard single-mode fiber (G652.A) [33] is stripped from its protective polymer buffer to enable the polishing of the end facet to an 8° angle. In the outline of this study, angle polishing is realized by employing a commercial fiber polishing machine (REV, KrellTech, Neptune City, NJ, USA) equipped with a bare fiber holder. This necessary procedure suppresses any unwanted back reflections of the fiber facet, and prevents the abundance of Fabry–Pérot resonances at the fiber-to-polymer junction. Subsequently, after filling the microstructure with a UV-curable adhesive (AC A535-AN, Addison Clear Wave Coatings, St. Charles, IL, USA), the fiber is inserted and aligned with the PPBG’s integrated waveguide. In the following, a UV light emitting diode is used to cure the adhesive. Finally, the lid’s microstructure is also filled with adhesive and, after mounting the lid on top of the sensor substrate, another UV curing step is executed. It is worthwhile to note that the capillary forces of the microstructure’s T-shaped design are exploited to prevent the adhesive from leaking between the sensor and lid, since this leads to immediate signal loss due to light coupling out from the waveguide. The maximum interconnection of adhesive, polymer and silica fiber is guaranteed by thermally post-curing the sensor at a temperature of about 85 °C for a duration of 1 h. When fully cured, the adhesive is specified to exhibit a glass transition temperature of 165 °C. A schematic of the microstructure-based fiber-to-chip coupling configuration, as well as a photograph of the sensor substrate and its respective lid, is shown in Figure 2. After its final assembly, the polymer sensor exhibits a length of 20 mm, a width of 10 mm and a thickness of 1.7 mm.

## 3. Results and Discussion

### 3.1. Lateral Misalignment

The coupling efficiency, η, of two waveguides is defined by the overlap integral of their electric field distributions, $\Psi_{1}$ and $\Psi_{2}$, as

(2) $$\eta =\frac{{\left|\int_{-\infty }^{+\infty }\Psi_{1}\left(x,y\right)\;\Psi_{2}\left(x,y\right)\mathrm{dx}\;\mathrm{dy}\right|}^{2}}{\int_{-\infty }^{+\infty }{\left|\Psi_{1}\right|}^{2}\mathrm{dx}\;\mathrm{dy}\;\int_{-\infty }^{+\infty }{\left|\Psi_{2}\right|}^{2}\mathrm{dx}\;\mathrm{dy}}.$$

**Figure 3:**
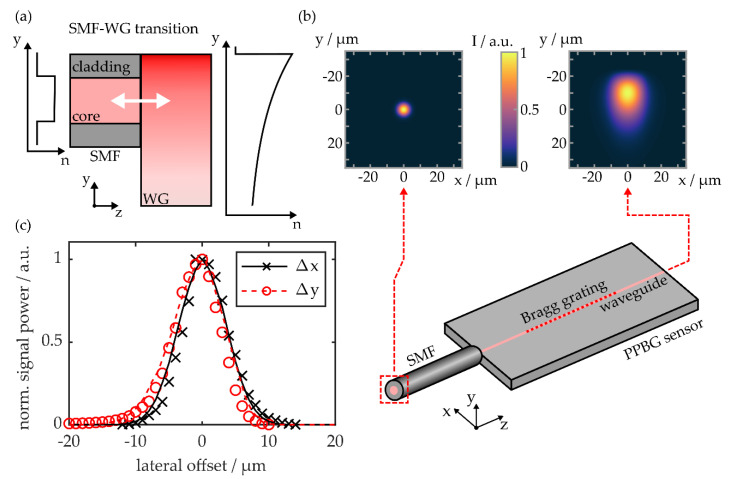
(**a**) Schematic of the SMF-to-WG butt coupling intersection and their respective refractive index profiles; (**b**) SMF-to-PPBG butt coupling and simulated fundamental mode intensity profiles of the SMF and the integrated waveguide; (**c**) PPBG signal power decrease due to lateral misalignment. The data points are acquired experimentally, whereas the depicted lines represent simulation results.

Light is defined to propagate along *z*, whereas the *x*- and *y*-axis are oriented parallelly or perpendicularly to the PPBG substrate surface, respectively. In case of a single-mode fiber, which features a rotationally symmetric refractive index profile, the fundamental mode’s intensity distribution is given by a Gaussian function. However, as shown in Figure 3a, integrated waveguides manufactured by the SWS technique exhibit an asymmetric and exponentially decaying refractive index depth profile in the *y*-direction. This, in turn, leads to an asymmetric intensity distribution of the waveguide’s fundamental mode perpendicular to the substrate surface. Assuming the separability of the asymmetric mode’s *x*- and *y*-components enables approximation by employing a Hermite–Gaussian or a combination of half-Gaussians, as demonstrated on waveguides within lithium niobate [34,35]. In the outline of this study, numerical simulation (RSoft, Synopsys, Mountain View, CA, USA) based on the beam propagation method [36] is used to support the experimental quantification of lateral misalignment losses. Figure 3b depicts a fundamental mode contour plot of a standard SMF, with a specified mode field diameter of 9.5 µm at a wavelength of 1550 nm. Additionally, the mode profile of an integrated PPBG waveguide, designed according to the fabrication parameters stated in Section 2, in combination with preliminary refractive index depth profile measurements, is given [37].

An orthogonally cleaved standard single-mode fiber is positioned on a high-precision translation stage (MAX313/M, Thorlabs, Bergkirchen, Germany) and aligned with the waveguide of a COC-based PPBG sensor (see Figure 3b). The polymer substrate does not feature a microstructure, in order to ensure that the fiber’s lateral positioning is not limited. To mitigate Fresnel reflections, an uncured optical adhesive (NOA76, Norland, Cranbury, NJ, USA) serves as the refractive index matching medium within the fiber-to-waveguide junction. Lateral and angular positioning is optimized towards the maximum reflection signal of the integrated Bragg grating, acquired by an industrial-grade interrogation unit (HYPERION si155, Micron Optics, Atlanta, GA, USA). While the PPBG’s position is fixed, the SMF is laterally offset in the *x*- or *y*-direction. The decline in detected signal power as a function of horizontal, Δx, and vertical offset, Δy, is shown in Figure 3c.

In the *x*-direction, the determined signal reduction is symmetric, whereas in the *y*-direction, the integrated waveguide’s asymmetric mode profile yields an asymmetric misalignment behavior. In both directions, the experimental data correlates well with simulated misalignment coupling losses, while it is worthwhile to note that the numerical model does not consider the impact of the refractive index matching medium or the necessary practical distance of fiber and waveguide in the z-direction. Based on the experimental data, an overall horizontal offset of ±8 µm leads to a signal loss of −10 dB, while vertically this is true for offset values of −9 µm and +6 µm, respectively. The findings underline the necessity of designing a robust coupling scheme, which protects the fiber-to-chip junction from environmental influences and thus prevents unacceptable reflection signal deteriorations in the field.

### 3.2. Pressure and Temperature Resistance

**Figure 4:**
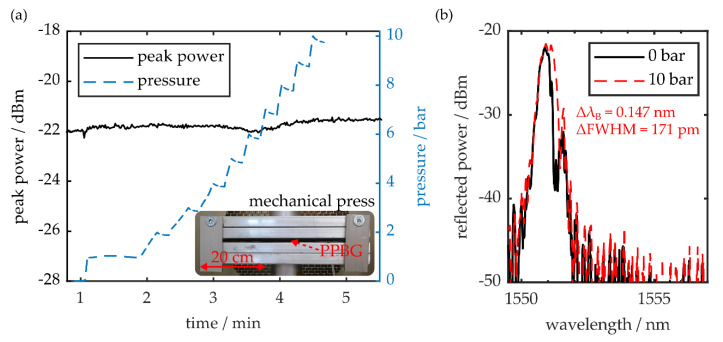
(**a**) Time trace of the PPBG’s peak signal power while the sensor is exposed to increasing pressure by a mechanical press. The inset shows the employed heated mechanical press. (**b**) Reflection peak without applied load and at a pressure of 10 bar.

In order to demonstrate the microstructure-based coupling scheme’s resistance to external pressures and elevated temperatures, the sensor is positioned between the metal stamps of a heated mechanical press. At first, the press is kept at room temperature while the applied load is incrementally increased with a speed of 1 mm·min^−1^. The in situ evaluation of the PPBG’s reflection signal is done by the same interrogator, whereas the applied pressure is actively controlled by a tensile testing machine (Autograph AG-X 20kN, Shimadzu, Duisburg, Germany). A time trace of the PPBG’s reflection peak power, up to a maximum pressure of 10 bar, is given in Figure 4a.

According to the observed results, the proposed fiber-to-chip coupling scheme is capable of withstanding pressures up to 10 bar without noticeable deterioration in signal strength. Additionally, besides a Bragg wavelength shift of 0.147 nm, the signal peak exhibits only minor shape deviations at maximum pressure, as shown in Figure 4b. The residual peak splitting, as well as the increase in spectral full width at half maximum (FWHM) of 171 pm, can be attributed to deformations of the polymer-based Bragg grating and stress-induced birefringence [38].

**Figure 5:**
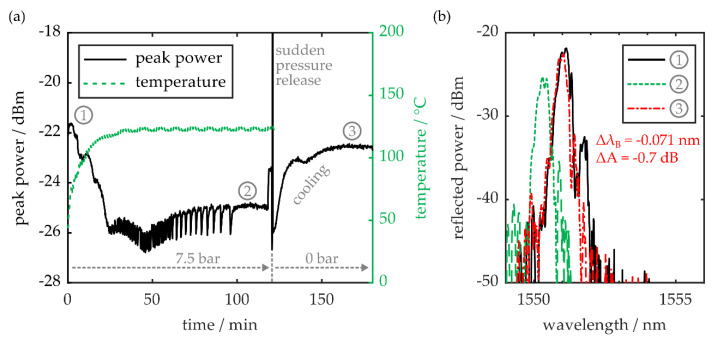
(**a**) Reflected peak power of the PPBG and temperature of the mechanic press during a heated pressure cycle. (**b**) Reflection spectra at room temperature and a pressure of 0 bar ①, at a temperature of 120 °C and a pressure of 7.5 bar ②, and after removal of the sensor from the heated press, cooled back down to room temperature ③.

In a second experimental cycle, the PPBG is exposed to a constant pressure of 7.5 bar for a duration of 2 h. Simultaneously, the mechanical press, which is positioned inside an air circulated furnace, is heated up to a temperature of 120 °C. Figure 5a shows the optical sensor’s reflection signal amplitude as well as the temperature within the air circulated furnace. Please note that the depicted temperature curve is acquired by evaluating two resistance thermometers, which are in physical contact with the upper and lower metallic stamp of the mechanical press.

While heating up, during the first 30 min of the cycle, a decrease in signal amplitude of −4 dB can be observed. Subsequently, the average temperature of the metallic stamps as well as the PPBG’s signal amplitude saturates, although there are still noticeable fluctuations. At the end of the cycle, pressure is suddenly released from the PPBG, which leads to a short but distinct amplitude fluctuation. Afterwards, the polymer sensor is removed from the heated press setup. During the subsequent cool-down period of 40 min, the detected signal power almost completely recovers. While there is negligible change in FWHM after the experiment, the Bragg signal peak exhibits a residual wavelength shift of −71 pm, as well as a peak power reduction of −0.7 dB. This leads to the conclusion that the observed amplitude variations and fluctuations are caused by a thermal coefficient of expansion mismatch between adhesive and COC substrate, which results in a misalignment of fiber and integrated waveguide. Amplitude fluctuations, observed while the setup is seemingly in thermal equilibrium, are caused by the active temperature control hysteresis of the air circulated furnace. However, this process cannot be resolved by the employed temperature probes since they are connected to the press’ metal stamps. Their comparably high mass prevents the detection of dynamic temperature variations, while the coupling junction is still directly exposed to the air stream generated by the furnace. The artifact observed during sudden pressure release, on the other hand, is attributed to a dynamic expansion mismatch of silica fiber, adhesive and polymer substrate. According to Figure 5b, which depicts the PPBG’s reflection spectra at the beginning of the experiment, at a temperature of 120 °C and a pressure of 7.5 bar and after the combined heat and pressure cycle, there are negligible peak distortions, even at elevated pressures and temperatures. Thus, the COC-based PPBG, as well as the proposed fiber-to-chip coupling scheme, are not only capable of withstanding environmental conditions this harsh, but the polymer-based photonic device also retains its functionality during the process.

### 3.3. Resistance to Tensile Forces

**Figure 6:**
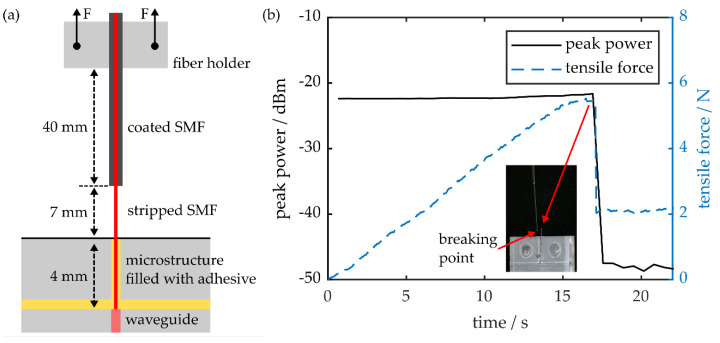
(**a**) Schematic of the employed tensile testing setup. (**b**) Time trace of the PPBG’s reflection peak power while the applied tensile force is gradually increased. Sudden signal loss occurs due to breakage of the stripped single-mode fiber, as depicted in the inset.

The robustness of the proposed coupling scheme to tensile forces is examined by mounting a fiber-coupled PPBG in a tensile testing machine (EZ-LX, Shimadzu). While the sensor’s positioning is fixed throughout the process, the distance between fiber mount, which is firmly attached to the single-mode fiber, and PPBG is gradually increased with a speed of 1 mm·min^−1^. This way, a tensile force is introduced into a fiber section of defined length, as well as the interconnection between silica fiber and planar polymer substrate. A schematic of the employed setup is given in Figure 6a, while Figure 6b depicts a time trace of the experimental results.

Up to a maximum value of 5.5 N, the applied tensile force increases linearly, whereas the observed signal amplitude remains constant. When reaching the curve’s yield point, sudden signal loss occurs due to fiber breakage in the stripped section of the SMF, close to the PPBG, as shown in the inset of Figure 6b. The brittle tensile behavior, as well as the resulting yield stress of 451 MPa, determined by correlating the maximum tensile force with the stripped SMF’s outer diameter of 125 µm, fits well with the preliminary data of fused silica fibers or rods [39,40]. Conclusively, the microstructure-based coupling scheme’s resistance to tensile forces is limited by the breaking point of the employed fiber.

### 3.4. Carbon Fiber Reinforced Polymer Integration

**Figure 7:**
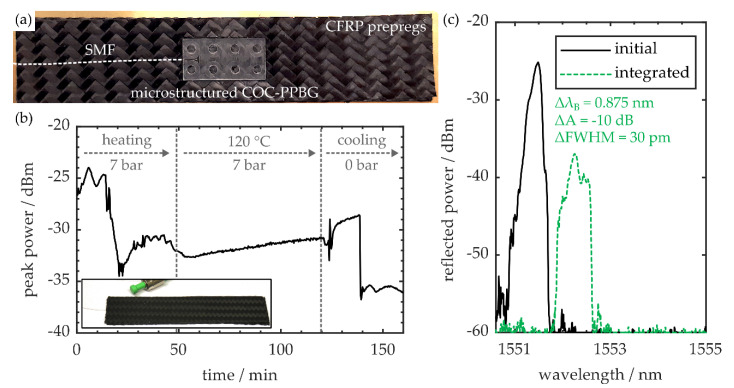
(**a**) COC-PPBG with a microstructure-based fiber-to-chip coupling scheme within an uncured carbon fiber reinforced polymer (CFRP) prepreg stack. (**b**) In situ monitoring of the PPBG’s peak signal power during the curing procedure. The inset shows the cured CFRP specimen with integrated polymer-optic sensor. (**c**) Reflection spectra of the PPBG prior to and after CFRP integration.

In total, 12 pre-impregnated composite fiber (prepreg) layers (P3252S-25, Toray Industries, Tokyo, Japan), each exhibiting a fiber orientation of ±45° and a thickness of 0.24 mm, are cut to rectangular shape with a length of 10 cm and a width of 2 cm. Eight layers are equipped with a sensor pocket, which is generated by cutting out the central section of the respective layer according to the sensor’s dimensions. Four of the employed prepregs remain unmodified, whereas two of them constitute the workpiece’s respective outermost layers. After assembling the carbon fiber reinforced polymer specimen’s bottom half, the PPBG is inserted into the sensor pocket as shown in Figure 7a. Subsequently, the workpiece is finalized by stacking the remaining prepregs on top. In order to prevent the CFRP matrix from sticking to the curing apparatus, the prepreg stack is sandwiched in-between two brass foils before the specimen is inserted into the heated mechanical press. The workpiece is then cured by exposing the prepreg stack to a pressure of 7 bar, at a temperature of 120 °C, for a duration of 2 h. Simultaneously, the amplitude of the PPBGs reflection signal is monitored. As depicted in Figure 7b, major amplitude reductions are observed during the heating and cooling of the specimen. Since these fluctuations are short-time events, they are attributed to dislocations of the fiber within the microstructure due to external forces. During heating, the compression of the CFRP workpiece, in combination with epoxy leaking from the rectangular specimen, leads to locally inhomogeneous pressure distributions.

After finishing the curing process, the specimen is removed from the mechanical press so it can cool down to room temperature. In the outline of this procedure, internal material tensions lead to workpiece deformations, and thus alternating stress or strain forces interacting with the embedded sensor and the fiber-to-chip junction [41]. The cured CFRP specimen with embedded PPBG, exhibiting a post-curing thickness of 2.6 mm, is also shown in Figure 7b, while the integrated polymer sensor’s reflection spectra, before and after curing, are shown in Figure 7c.

Besides a signal amplitude reduction of −10 dB, the embedded PPBG also exhibits a Bragg wavelength shift of 0.875 nm and an increase in spectral FWHM of 30 pm. According to Section 3.1, this magnitude of signal loss correlates to a maximum lateral fiber-to-waveguide misalignment of 9 µm. The change of the latter two signal parameters underlines that the sensor constantly experiences residual stress by the cured surrounding CFRP. Although the COC-PPBG experiences noticeable losses in signal strength after curing, the reflection peak still exhibits a dynamic range of 24 dB, and can thus be utilized for sensing purposes. Therefore, the proposed microstructure-based fiber-to-chip coupling scheme enables the position-independent integration of polymer planar Bragg gratings into commercial-grade carbon fiber reinforced polymer workpieces.

## 4. Conclusions

In summary, this study proposes and demonstrates an outstandingly robust microstructure-based fiber-to-chip coupling scheme for polymer planar Bragg grating devices. Every PPBG consists of two platelets, which are macro- and microstructured out of an injection-molded cyclic olefin copolymer by means of a milling process. One of the platelets serves as substrate for the photonic sensing platform while the other is used as a protective lid in order to shield the fiber-to-chip junction from environmental influences. A physical connection between silica fiber and optical polymer is established by means of a high-grade UV-curable adhesive. Multiple experimental cycles in a heated mechanical press reveal that, at room temperature, the proposed coupling scheme is able to withstand pressures up to 10 bar, without any sensor signal deterioration. The simultaneous exposure of the fabricated polymer sensor to a pressure of 7.5 bar and a temperature of 120 °C, for a duration of 2 h, yields noticeable signal amplitude fluctuations up to 4 dB. However, the PPBG’s reflection signal recovers after the experimental cycle, which leads to the conclusion that the observed fluctuations are caused by thermal expansion mismatches arising in the coupling region. Examination of the developed coupling scheme’s robustness to tensile loads shows that it completely withstands tensile forces up to the yield strength of the employed silica fiber, whereas fiber breakage occurs at a tensile stress of 451 MPa. Finally, an application example is given by completely integrating the microstructured PPBG into a commercial-grade carbon fiber reinforced polymer specimen. After its integration, the sensor signal still exhibits a strong reflection peak with a dynamic range of 24 dB, and can thus be used for sensing purposes. Therefore, the demonstrated microstructure-based fiber-to-chip coupling approach is capable of paving the way towards practical harsh-environment strain and temperature sensing applications, not only for polymer-based sensors, but for planar photonic devices in general.

## Figures and Tables

**Table 1:** Overview of the employed milling tools and parameters.

	Macrostructures	Microstructures
tool diameter/mm	1 ^1^	0.254 ^2^
spindle speed/min^−1^	15,000	30,000
feed rate/mm·min^−1^	500	500
cross infeed/mm	0.20	0.04
depth infeed/mm	0.25	0.04

^1^ (ES-PS-0100-3-040-40, vhf camfacture, Ammerbuch, Germany); ^2^ (TS-2-0100-S, Performance Micro Tool, Janesville, WI, USA).
